# Shaping the human face: Periosteal bone modeling across ontogeny

**DOI:** 10.1002/ar.25689

**Published:** 2025-05-19

**Authors:** Sarah E. Freidline, Madison Hubbart, Catherine Shipman, Najielie Burgos, Chiara Villa, Alexandra Schuh

**Affiliations:** ^1^ Department of Anthropology University of Central Florida Orlando Florida USA; ^2^ Department of Human Origins Max Planck Institute for Evolutionary Anthropology Leipzig Germany; ^3^ Department of Forensic Medicine University of Copenhagen Copenhagen Denmark

**Keywords:** bone modeling, bone resorption, geometric morphometrics, growth and development, human evolution

## Abstract

Facial morphology is a defining aspect of *Homo sapiens* that distinguishes our species from fossil ancestors and plays a central role in estimating age, sex, and ancestry in both past and present populations. Understanding how the face develops during postnatal ontogeny is essential for interpreting adult facial variation. Periosteal bone modeling (i.e., patterns of resorption and formation) provides direct evidence of bone growth activity underlying morphological variation. This study quantifies periosteal bone modeling in a cross‐sectional ontogenetic sample of individuals ranging from birth to adulthood from three geographical populations: Western Europe, Greenland, and South Africa. Epoxy replicas were analyzed using digital microscopy to quantify bone resorption, and digital maps of the bone modeling patterns were created for each facial region—brow ridge, zygomatic, maxilla, and mandible—and projected onto three‐dimensional surface models. In parallel, geometric morphometric and multivariate statistical analyses were used to evaluate ontogenetic patterns. Results highlight a consistent sequence of resorption and deposition during human ontogeny and a strong pattern of covariation between bone modeling and shape for most facial regions. The face is largely resorptive from early ontogeny, with deposition increasing with age; the maxilla is significantly more resorptive than other facial regions. Greater resorption in the midface corresponds to significant facial growth and development in early ontogeny, and a developmental shift around adolescence marks the transition from primarily downward to more forward‐oriented growth. Overall, the combined approach underscores the developmental coordination of the face and suggests that the human facial growth pattern reflects the need to maintain a non‐projecting face from birth on.

## 1 INTRODUCTION

Facial morphology is a defining characteristic of *Homo sapiens*, distinguishing us from both our fossil ancestors and other primates (Stringer, 2016). It also plays a central role in age, sex, and ancestry estimation in both past and present human populations (Buikstra & Ubelaker, 1994). However, the extent to which postnatal ontogeny shapes adult facial morphology remains unclear. Adult craniofacial form results from a complex interplay of pre‐ and postnatal developmental processes influenced by genetic, environmental, and cultural factors, including climate, diet, and social practices (e.g., Hallgrimsson et al., 2007; Hubbe et al., 2009; Lieberman, 2011; von Cramon‐Taubadel, 2014). Because facial bones are among the last components of the skull to complete growth, they are particularly sensitive to these influences. An increasing number of studies suggest that differences in adult morphology, both at the species level and the population level, arise prenatally or during early postnatal development and become more pronounced throughout postnatal growth (Ackermann & Krovitz, 2002; Bastir et al., 2007; de Ponce León & Zollikofer, 2001; Freidline et al., 2015; Strand Viðarsdóttir et al., 2002). Understanding the timing and processes of postnatal facial development is essential for interpreting variation in both modern and extinct human populations.

Craniofacial development involves the dynamic process of three distinct but interrelated mechanisms: cortical drift, bone displacement, and rotation (Enlow & Hans, 2008; Lieberman, 2011). Cortical drift involves the localized reshaping of bone surfaces through the process of bone modeling, the asynchronous activities of osteoblasts (bone formation) and osteoclasts (bone resorption). This activity results in “drift” of the bone's cortical surface, allowing it to change shape and size without altering its overall spatial position within the skeleton. Bone displacement describes the movement of entire bones due to growth at sutures, synchondroses, or other growth centers, and rotation ensures proper alignment in response to growth and mechanical demands. Collectively, these processes guide the development of craniofacial bones, ensuring structural and functional integrity during growth.

Compared to non‐human primates and fossil hominins, human faces are retracted under the anterior cranial base and are more orthognathic (i.e., flat). The development of the chin, a uniquely human trait, is often linked to facial retraction. These features evolved at different rates within our species in the last 300,000 years (Hublin et al., 2017) and have been shown to be related to the evolution of neurocranial globularity and increased cranial base flexion (Lieberman et al., 2002; Zollikofer et al., 2022). Much of our knowledge regarding human facial growth is from the seminal works of Donald Enlow, who identified that the development of the adult human face reflects temporal changes in bone modeling (i.e., bone formation and resorption) during ontogeny (Enlow, 1962; Enlow, 1963, 1966, 1990; Enlow & Bang, 1965; Enlow & Hans, 1996; Enlow & Hans, 2008; Enlow & Harris, 1964; Enlow & Moyers, 1971). Histological studies, first carried out by Enlow and later expanded on by others, have shown that bone resorption during ontogeny limits forward facial growth, explaining our orthognathic faces (e.g., Brachetta‐Aporta et al., 2019b; Brachetta‐Aporta et al., 2021; Bromage, 1989; Enlow & Hans, 2008; Kurihara et al., 1980; Martinez‐Maza et al., 2013; McCollum, 2008; Schuh et al., 2019; Schuh et al., 2020). Further work on human facial growth by Martinez‐Maza et al. (2013) found that adults exhibit greater bone formation surfaces compared to subadults, reflecting a developmental shift in the facial growth trajectory from predominantly downward growth in subadults to forward growth in adults. These ontogenetic changes accommodate physiological and functional demands, such as enlarging the oral and nasal cavities after the cessation of growth and the closure of cranial sutures (Enlow & Hans, 2008; Moss & Young, 1960). While these studies have been instrumental in advancing the understanding of human facial growth and development, they often lack quantitative data and rely on small sample sizes with incomplete ontogenetic age representation.

Geometric morphometric (GM) shape analysis and three‐dimensional (3D) imaging have further enriched our understanding of macroscopic facial growth, offering insights into the dynamic ontogenetic processes shaping human facial morphology (e.g., Barbeito‐Andrés et al., 2011; Bulygina et al., 2006; Coquerelle et al., 2011; Freidline et al., 2015; Klop et al., 2024; Rosas & Bastir, 2002; Strand Viðarsdóttir et al., 2002). GM methods complement histological studies as they allow researchers to visualize and quantify shape changes and movements in bone resulting from bone modeling activity, displacements, and rotations. Recent work has been underway to develop a framework for integrating GM shape analysis and the cellular processes of bone growth (e.g., Brachetta‐Aporta et al., 2019a, 2019b; Brachetta‐Aporta et al., 2021; Freidline et al., 2017; Martinez‐Maza et al., 2015; Schuh et al., 2019; Schuh et al., 2020; Schuh et al., 2021). Methodologically, these studies have successfully quantified periosteal bone deposition and resorption and its covariation with bone shape, enabling a statistical framework for interpreting ontogenetic patterns in bone modeling, while also providing powerful visualizations to aid in the interpretation of bone modeling and shape patterns. The resulting bone modeling maps generated from these studies enable comparisons across sample populations and during ontogeny, shedding light on bone turnover during postnatal ontogeny. Together, these studies have shown that overall middle (maxilla and zygomatic) and upper (brow ridge) facial growth is highly constrained across diverse human populations and throughout ontogeny, with some differences arising during postnatal ontogeny possibly related to differences in diet or population history (Brachetta‐Aporta et al., 2019a, 2019b; Brachetta‐Aporta et al., 2021; Schuh et al., 2019; Schuh et al., 2020). These findings have also shown that facial morphology is driven by changes in osteoblastic and osteoclastic rates of expression, rather than differences in bone modeling patterns (i.e., changes in location of formation and resorption). However, the limited studies that have applied these methods have focused on the middle and upper face, excluding the lower face (mandible). Additionally, small sample sizes and the omission of the youngest individuals (prior to the first permanent molar eruption) have led to an incomplete representation of ontogeny.

Building on previous research, this study combines surface histology and GM shape analyses to examine the entire facial skeleton, including the mandible, as a dynamic unit. In doing so, this study quantifies and visualizes ontogenetic bone modeling patterns in the facial skeletons of a geographically diverse sample of recent humans, incorporating individuals across the full ontogenetic timeline. The objectives of this study are to (1) identify temporal patterns in the expression of resorption on the periosteal bone surface between different facial regions; (2) compare bone turnover to facial growth rates and related morphological changes; and (3) test patterns of covariation between microscopic bone modeling patterns and macroscopic shape changes. This integrative approach not only enhances our knowledge of facial ontogeny but also establishes a foundation for comparative research across species.

## 2 MATERIALS AND METHODS

### 2.1 Sample

The sample is composed of individuals from three distinct geographical regions: Western Europe (Anatomical Institute of Strasbourg, France; University of Leipzig Anatomical Collection, Germany); Greenland (Greenland National Museum and Archives); and South Africa (Iziko South African Museum, Cape Town; McGregor Museum, Kimberley; and University of Cape Town). All human remains were handled in accordance with ethical guidelines, and appropriate permissions were obtained from the curating institutions prior to data collection. These populations were chosen because of their diverse geographic locations and lifestyles and broad range of facial morphologies (Freidline et al., 2015; Strand Viðarsdóttir et al., 2002). The Western European population lived in a temperate, oceanic climate during the Industrial Era; the Greenlandic Inuit population were Arctic, marine hunter‐gatherers; and the South African San and/or Khoe were also hunter‐gatherers but living in a warmer and more temperate environment. A broad sample is essential to capture the diversity in facial morphology among present‐day humans, as our goal is to identify patterns at the species level (the human pattern). Diet is particularly relevant to the discussion since muscles are among the first to respond to changes in food properties, which may subsequently influence bone morphology. However, we acknowledge that our sample size is too small to perform separate statistical analyses for population differences and that additional methods are better to more directly test correlations with climate and diet. The ontogenetic age of the individuals ranges from birth to adulthood. The Western European population is the only group in the study with known age and sex. The age for the Greenlandic Inuit and South African groups were previously estimated according to dental development by Schuh et al. (2020), following the criteria of AlQahtani et al. (2010). Sex for these two populations was never estimated due to difficulties in juvenile sex estimation. All individuals were classified into five age groups according to dental development: no teeth erupted (AG 1, or neonates); developing deciduous dentition, until completion (AG 2); first permanent molar (M^1^) fully erupted (AG 3); second permanent molar (M^2^) fully erupted (AG 4); third permanent molar (M^3^) fully erupted (or adult; AG 5). Efforts were made to ensure an equal distribution across age groups and populations (see Supplementary Tables 1 and 2); however, some imbalances were unavoidable. Specifically, the AG 1 sample predominantly consists of individuals of Western European origin, while AG 4 lacks representation from this population. To increase sample size, populations were pooled according to age groups. Consequently, the sample size varies from 35 to 51 individuals, depending on the specific bone and analysis (Tables 1 and 2).

**TABLE 1:** Number of individuals attributed to each age group (AG) for each facial region in the bone modeling analysis.

Region	AG 1	AG 2	AG 3	AG 4	AG 5	Total
Brow ridge	5	15	13	4	9	46
Zygomatic	4	15	13	5	9	46
Maxilla	4	20	14	5	8	51
Mandible	7	14	11	3	6	41

*Note*: Further details regarding group composition can be found in Supplementary Table 1. AG 1: No teeth erupted (neonate); AG 2: Developing deciduous dentition, until completion; AG 3: First permanent molar (M^1^) fully erupted; AG 4: Second permanent molar (M^2^) fully erupted; AG 5: Third permanent molar (M^3^) fully erupted (adult).

**TABLE 2:** Number of individuals attributed to each age group (AG) for the mid and upper face (composed of brow ridge, maxilla, zygomatic) and mandible (composed of internal and external mandibular datasets) in the geometric morphometric analyses.

Region	AG 1	AG 2	AG 3	AG 4	AG 5	Total
Mid/upper face	4	13	9	4	5	35
Lower Face	4	13	10	5	6	38

*Note*: Further details regarding group composition can be found in Supplementary Table 2.

### 2.2 Data acquisition and analysis

When possible, bone modeling and GM data were collected from each individual. 3D surface models were digitally reconstructed from either computed tomographic (CT) scans (voxel size resolutions between 0.06 and 0.2 mm) using Avizo v. 7.1 (FEI Visualization Sciences Group, Hillboro), or when CT data was not available, surface scans using the portable Arctec Space Spider (Arctec3D, voxel size resolution: 0.1 mm).

**FIGURE 1:**
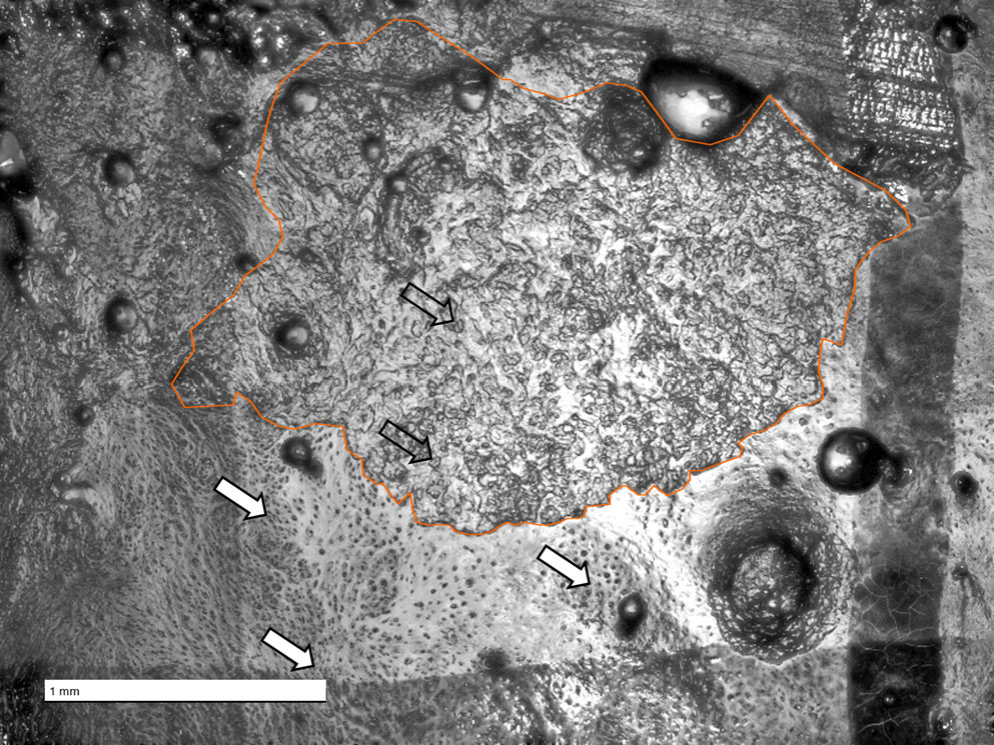
Example of bone formation and resorption on a positive replica. The faint black lines outline the 2.5 mm square drawn on the replica. Solid white arrows point at the collagen fiber bundles, indicative of bone formation. Open arrows point at Howship's lacunae. Orange outline represents a resorptive area selected in ImageJ. Scale bar: 1 mm.

**FIGURE 2:**
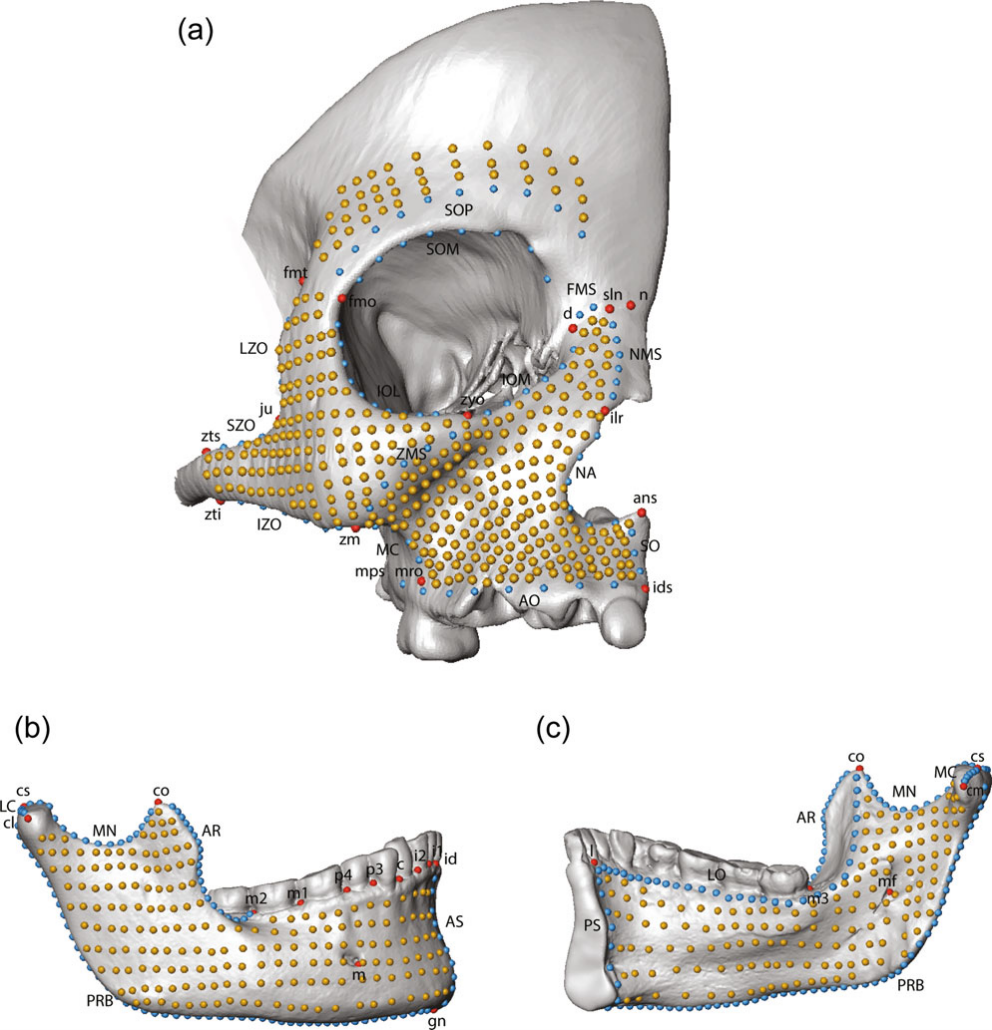
Landmarks (red), curve semilandmarks (blue), and surface semilandmarks (yellow) used in the geometric morphometric analyses: (a) upper and middle face, (b) lower face external, and (c) lower face internal. Landmark and curve definitions provided in Table 3. Landmarks subsets are illustrated in Supplementary Figure 1.

*Bone modeling analysis*: Replicas of the periosteal surface of the face were made using low‐viscosity silicone (President Plus light body, Coltène/Whaledent AG, Switzerland) following Bromage (1985). Silicone molds of the upper face (brow ridge), middle face (zygomatic and maxilla), and the lower face (mandible) were made. The nasal bones were not studied due to their infrequent preservation in archeological samples. Both the external (buccal and labial) and internal (lingual) periosteal surfaces of the mandible were replicated. High‐resolution positive replicas were then obtained by applying a transparent epoxy resin (5 Min Epoxy Epoxidharz 2 L‐Kleber transparent, Devcon; J‐B Weld Ultrarez UV‐Resistant Coating & Casting Epoxy). Only the best‐preserved side of the face (left or right) was analyzed. Following the protocol outlined in Schuh et al., (2019), a 5 × 5 mm grid was drawn on each positive replica. Each square of the grid was viewed using a digital optical microscope (Smart Zoom 5, Zeiss) using a 5× PlanApo D objective (zoom: 101×) to identify bone resorption and formation (Figure 1). Bone resorption is visible as cavities on the bone surface, called Howship's lacunae, produced by acidic dissolution (Boyde, 1972). A *resorptive area* refers to a region of the bone surface where these lacunae are clustered, indicating active or past osteoclastic activity. Bone formation is identified as mineralized collagen fiber bundles produced by osteoblasts. When both bone formation and resorption were identified in a square, a new grid of 2.5 mm × 2.5 mm squares was drawn within the 5 × 5 mm square, and pictures of the sub‐squares were made cf., Figure 2 Schuh et al., (2019). In line with previous studies (Bromage, 1989; Lacruz et al., 2013; Lacruz, Bromage, O'Higgins, Arsuaga, et al., 2015; Lacruz, Bromage, O'Higgins, Toro‐Ibacache, et al., 2015; Martinez‐Maza et al., 2011; McCollum, 1999, 2008; McCollum & Ward, 1997; Rosas & Martinez‐Maza, 2010), we did not distinguish between highly active and quiescent bone activity as resting surfaces are challenging to identify and quantify on dry bone and the primary objective of this study was to record the location of each cellular activity on the bone surface.

Resorptive areas were manually selected on each image using the polygon selection tool in ImageJ (Figure 1; Schneider et al., 2012). The amount of bone resorption was calculated by summing all areas of bone resorption, dividing by the surface area of the bone or region, and converting the result into a percentage (% BR) by multiplying by 100. To obtain the total %BR for the different facial regions (i.e., brow ridge, zygomatic, maxilla, external mandible, and internal mandible), the bone resorption areas from each cast were summed and corrected by size (determined by the total area of the region). This value was calculated independently for each facial region and used to study the variation in bone resorption in each age group. We performed a Type III analysis of variance (ANOVA) to evaluate the effects of facial region (independent variable) on resorption (dependent variable). Type III ANOVA was selected because it accounts for unequal sample sizes. Post hoc comparisons were conducted using Tukey HSD to determine which facial regions differ significantly in the amount of resorption.

Percentages of bone resorption were used for the computation of the digital bone modeling maps for each individual in R Studio v. 2024.04.02 (R Core Team, 2024) and projected onto their respective surface model in Geomagic Studio v.2014 (Geomagic Inc., Research Triangle Park, USA). Squares showing only bone formation (i.e., no bone resorption) were coded as 0%, and squares showing only bone resorption were coded as 100%. To compare the changes in bone modeling and shape between species, the average bone modeling maps were projected onto the corresponding mean shapes for each age group. Samples were selected based on good surface preservation to minimize missing data. Although minimal, some missing data was inevitable. Missing data were coded as NAs and excluded from the computation of the mean maps for each age group. If missing data was found within one square together with either bone formation or bone resorption, the entire square was considered either fully forming or fully resorbing, as similar cellular activities are more likely to occur in neighboring areas (Brachetta‐Aporta et al., 2018).

*GManalysis*: 3D landmark and semilandmark coordinates were digitized on the right face of the surface models using Landmark Editor v.3.0.0.6 (Wiley et al., 2005) (Figure 2; Table 3). Type I landmarks were selected to anchor key, homologous anatomical points, while curve and surface semilandmarks were used to capture subtle shape variation in regions lacking discrete anatomical landmarks, particularly those corresponding to the areas of bone modeling; this combined approach ensured both anatomical precision and comprehensive surface coverage relevant to the study's aims. Curve and surface semilandmarks were slid by minimizing the bending energy of a thin‐plate‐spline deformation between each specimen and the sample mean shape (Gunz et al., 2005; Gunz & Mitteroecker, 2013). After sliding, all landmarks and semilandmarks were converted to shape variables using a generalized Procrustes analysis (Rohlf & Slice, 1990). Centroid size, the square root of the sum of squared distances from each landmark to the specimen's centroid, was used as a proxy for size (Dryden & Mardia, 1998). All data processing and analysis was done in RStudio v. 2024.04.02 (R Core Team, 2024), primarily using the Morpho (Schlager, 2017) and geomorph (Adams et al., 2021; Baken et al., 2021) packages.

**TABLE 3:** Landmarks and semilandmarks used in the geometric morphometric analyses. Landmarks and curve semilandmarks are labeled on Figure 2 and Supplementary Figure 1.

Landmark		Subset	
Nasion	n	Brow ridge, Maxilla	
Superolateral nasion	sln	Maxilla	
Dacryon	d	Brow ridge, Maxilla	
Zygoorbitale	zyo	Maxilla, Zyomatic	
Inferolateral nasion	Ilr	Maxilla	
Anterior nasal spine	ans	Maxilla	
Alveolare (infradentale superius)	ids	Maxilla	
Malar root origin	mro	Maxilla	
Zygomaxillare	zm	Maxilla, Zygomatic	
Maxillo‐palatine suture	mps	Maxilla	
Frontomalare orbitale	fmo	Brow ridge, Zygomatic	
Frontomalare temporale	fmt	Brow ridge, Zygomatic	
Zygomatic temporal suture superior	zts	Zygomatic	
Zygomatic temporal suture inferior	zti	Zygomatic	
Jugale	ju	Zygomatic	
Infradentale	id	External mandible	
Linguale	l	Internal mandible	
Gnathion	gn	External, Internal mandible	
Mentale	m	External mandible	
Mandibular foramen	mf	Internal mandible	
Coronoid process	co	External, Internal mandible	
Condylion laterale	cl	External mandible	
Condylion mediale	cm	Internal mandible	
Condylion superior	cs	External, Internal mandible	
Third molar	m3	External mandible	
Second molar	m2	External mandible	
First molar	m1	External mandible	
Canine	c	External mandible	
Second Incisor	i2	External mandible	
First Incisor	i1	External mandible	

Curve semilandmarks		Subset	Definition
Fronto‐maxillary suture (*n* = 2)	FMS	Brow ridge, maxilla	Superolateral nasion to dacyron
Naso‐maxillary suture (*n* = 6)	NMS	Maxilla	Superolateral nasion to inferolateral rhinion
Inferior orbital margin medial (*n* = 6)	IOM	Maxilla	Dacryon to zygorbitale
Nasal aperture outline (*n* = 6)	NA	Maxilla	Inferolateral rhinion to anterior nasal spine
Alveolar outline (*n* = 6)	AO	Maxilla	Alveolare to first molar
Subnasal outline (*n* = 3)	SO	Maxilla	Alveolare to nasospinale
Zygomatic‐maxillary suture (*n* = 5)	ZMS	Maxilla, zygomatic	Zygoorbitale to zygomaxillare
Maxillary contour (*n* = 4)	MC	Maxilla	Zygomaxillare to malar root origin
Superior zygomatic outline (*n* = 3)	SZO	Zygomatic	Jugale to zygomatic temporal suture superior
Inferior zygomatic outline (*n* = 6)	IZO	Zygomatic	Zygomaxillare to zygomatic temporal suture inferior
Lateral zygomatic outline (*n* = 3)	LZR	Zygomatic	Jugale to frontomalare temporale
Inferior orbital margin lateral (*n* = 10)	IOL	Zygomatic	Zygomaxillare to frontomalare orbitale
Superior orbital margin (*n* = 8)	SOM	Brow ridge	Frontomalare to dacryon
Supraorbital profile (*n* = 10)	SOP	Brow ridge	Frontomalare to nasion
Anterior symphysis (*n* = 10)	AS	External mandible	Infradentale to gnathion
Posterior symphysis (*n* = 10)	PS	Internal mandible	Linguale to gnathion
Anterior ramus (*n* = 20)	AR	External and internal mandible	Coronoid to M2
Lingual outline (*n* = 30)	LO	Internal mandible	Coronoid to infradentale
Mandibular notch (*n* = 15)	MN	External and internal mandible	Coronoid to condylion superior
Medial condyle (*n* = 5)	MC	Internal mandible	Condylion superior to condylion mediale
Lateral condyle (*n* = 5)	LC	External mandible	Condylion superior to condylion laterale
Posterior ramus and base (*n* = 50)	PRB	External and internal mandible	Condylion superior to gnathion

Surface semilandmarks			
Maxillary patch (*n* = 200)			
Zygomatic patch (*n* = 119)			
Brow ridge patch (*n* = 41)			
Mandible patch internal (*n* = 146)			
Mandible patch external (*n* = 189)			

*Note*: Landmark subsets are specified in the table and shown in Supplementary Figure 1. Definitions for curves are provided in the table.

To compare the relative amount of growth and development between facial regions, the upper and middle face were divided into three different landmark subsets: brow ridge, zygomatic, and maxilla (Table 3; Supplementary Figure 1) and an independent GPA was performed on each subset. Centroid size and Procrustes shape coordinates were used to quantify facial growth and development, respectively, where growth is defined as the relationship between ontogenetic stage and size and development as the relationship between ontogenetic stage and shape (Zollikofer & de Ponce León, 2004). Growth was calculated for each region of the upper and middle face and the mandible by comparing average centroid size differences between age groups (Freidline et al., 2015). Development was calculated in the same manner but using Procrustes distances instead of centroid size. Permutation tests (1000 iterations) were conducted to evaluate significant differences in size and shape between age groups, with significance determined at a threshold of 0.05.

To visualize developmental shape changes during ontogeny, the upper and middle facial skeleton were evaluated as a single unit and the lower face (mandible) as a single unit. Mean shapes were calculated for each age group (AG1 through 5) using both the upper/middle and lower face landmark datasets (Figure 2). Shape changes between age groups were visualized by superimposing the mean shape of the subsequent age group.

A two‐block partial least squares analysis (PLS) was performed to test the covariation between bone modeling and facial development (Brachetta‐Aporta et al., 2021; Rohlf & Corti, 2000; Schuh et al., 2019; Schuh et al., 2020). To do so, separate PLS analyses were performed for each region of the upper and middle face as well as the external and internal mandible, with the bone modeling map representing one block and the corresponding Procrustes shape coordinates representing the other (Supplementary Figure 1). For the mandibular analyses, the landmarks were divided into external and internal datasets to match the surface replicas, and independent GPAs were performed prior to the PLS analysis. Results were visualized by computing extreme shapes and textures (i.e., maps) on each axis ± two standard deviations from the mean.

## 3 RESULTS

### 3.1 Bone modeling analyses

**FIGURE 3:**
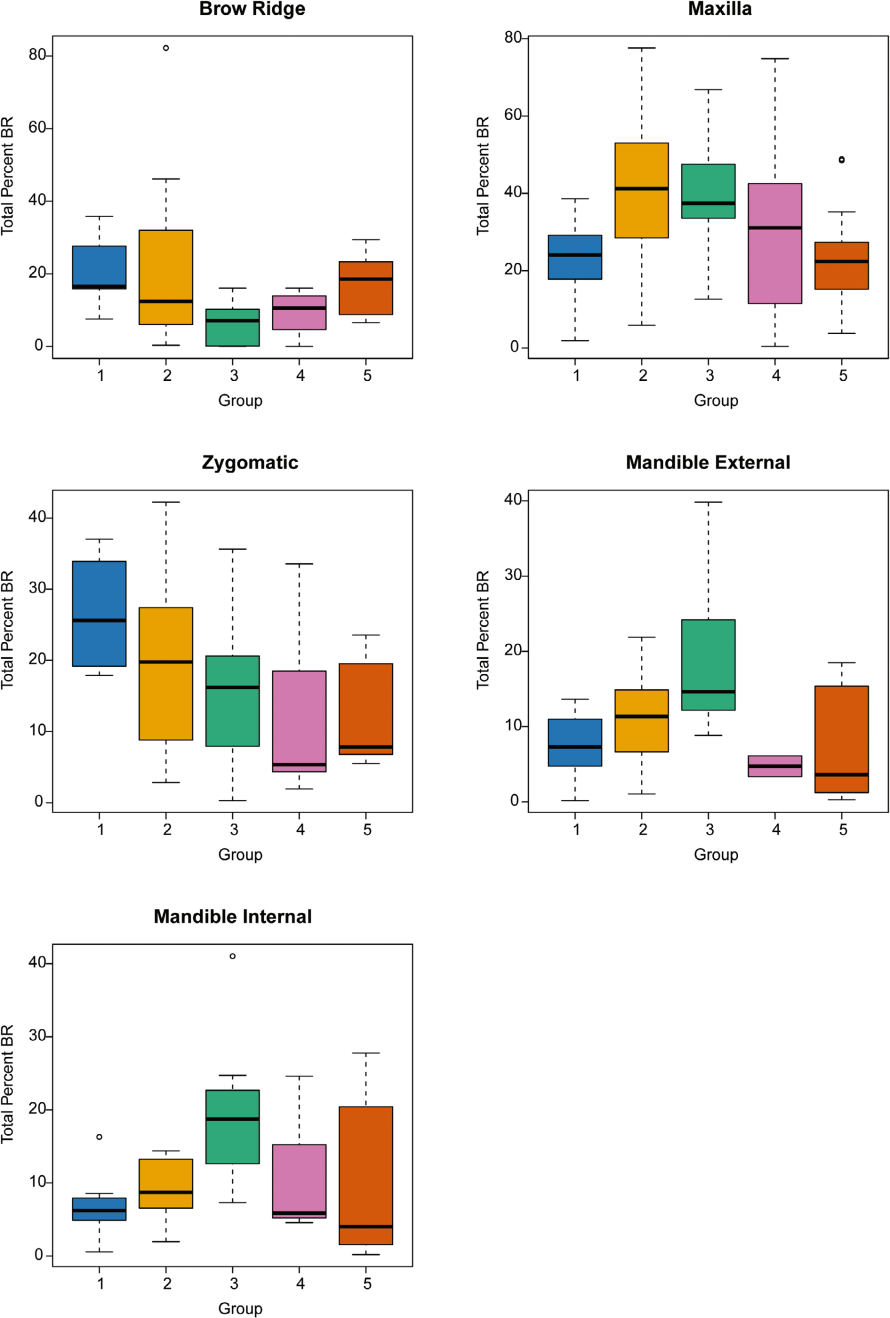
Boxplot of percentages of bone resorption for each facial region according to age group (AG 1 through 5). Horizontal lines represent the median of each age group. Boxes show the interquartile range (IQR, 25th to 75th percentile). Whiskers extend to 1.5 times IQR. Open circles are outliers.

*Expression of bone resorption during ontogeny*: The expression of bone resorption is most variable in the maxilla (Table 4, Figure 3). The maxilla has the highest mean percentage of bone resorption (35.2% of total bone area), significantly exceeding all other facial regions (*p‐*value: <0.001; Table 5). It is followed by the zygomatic (17.1%), brow ridge (15.1%), and mandible (11.7% internal and 11.4% external). Bone resorption in the zygomatic and brow ridge decreased from AG 1 to AG 3, while in the maxilla and mandible, it increased during these stages, peaking in AG 2 and AG 3, respectively, before declining in AG 4 and AG 5 (Figure 3).

**TABLE 4:** Percentages of bone resorption across facial regions.

Region	AG 1	AG 2	AG 3	AG 4	AG 5	Pooled AG mean
Brow ridge	16.5	12.4	7.1	10.6	18.5	15.1 (11.3)
Maxilla	24.1	41.2	37.5	31.1	22.4	35.2 (16.5)
Zygomatic	25.6	19.8	16.2	5.3	7.8	17.1 (11.1)
Mandible						
External	7.3	11.3	14.6	4.7	3.6	11.4 (8.1)
Internal	6.2	8.7	18.7	5.9	4.0	11.7 (8.6)

*Note*: Median resorption percentages (relative to total bone/region) are reported for each age group (AG), while mean resorption percentages (relative to total bone/region) are provided for each bone/region across all age groups, with standard deviations in parentheses.

**TABLE 5:** ANOVA results for pairwise comparisons in mean percentages of resorption for each facial region.

	Diff	Lwr	Upr	*p* adjusted
Maxilla‐brow ridge	20.13	13.50	26.76	***p* < 0.001**
Mandible external‐brow ridge	−3.67	−12.09	4.75	0.69
Mandible internal‐brow ridge	−3.46	−11.93	5.02	0.72
Zygomatic‐brow ridge	1.95	−6.17	10.07	0.95
Mandible external‐maxilla	−23.80	−30.79	−16.81	***p* < 0.001**
Mandible internal‐maxilla	−23.59	−30.65	−16.52	***p* < 0.001**
Zygomatic‐maxilla	−18.18	−24.81	−11.55	***p* < 0.001**
Mandible internal–mandible external	0.21	−8.55	8.98	1.00
Zygomatic‐ mandible. external	5.62	−2.79	14.04	0.25
Zygomatic‐ mandible. internal	5.41	−3.07	13.89	0.29

*Note*: significant values (*p* < 0.05) are highlighted in bold.

**FIGURE 4:**
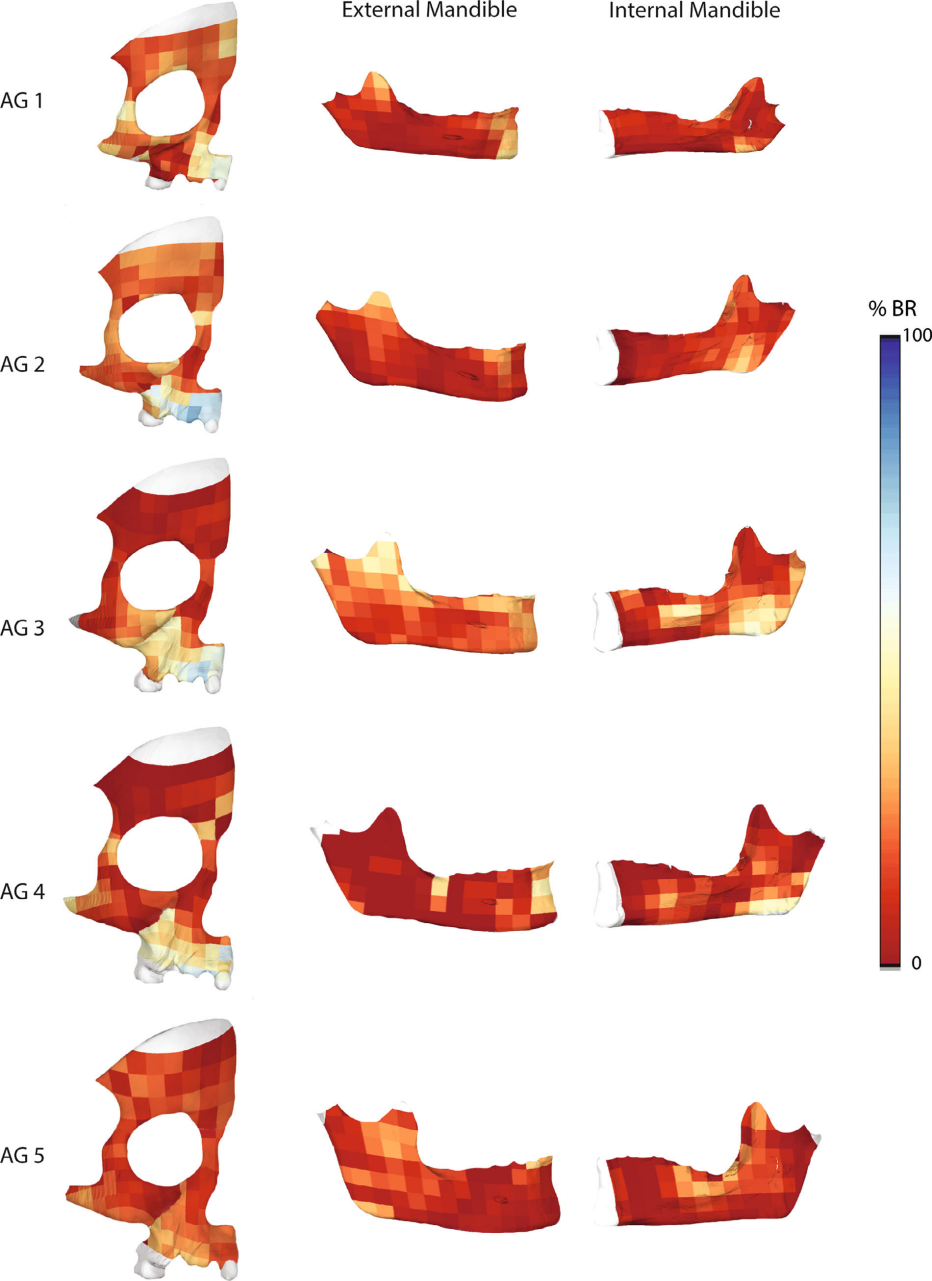
Mean bone modeling maps for each age group represented as heat maps and projected onto their corresponding mean shapes. High percentages of bone resorption (%BR) are shown in cold tones, while warm tones represent low %BR (i.e., where bone formation is predominant). Data were not collected from the mandibular condyle, which has been removed from the image.

*Bone modeling pattern during ontogeny*: Throughout ontogeny, the face is primarily depository, with exceptions in specific areas (Figure 4). In AG 1, bone formation is most common along the lateral brow ridge, posterior maxillary body, and frontal process of the maxilla, while resorption is concentrated on the medial brow ridge above the glabella, anterior maxilla, and the frontal and temporal processes of the zygomatic. In the mandible, resorption occurs externally at the coronoid process and along the symphysis at the incisal alveolar region and mentum osseum (chin), and internally at the gonial region.

In AG 2, the upper face and zygomatic are largely characterized by bone formation. Resorption is present along the maxillary alveolar process, as well as the frontomaxillary and zygomaxillary sutures. The mandible follows a similar pattern to the previous age group (AG 1) but shows little to no resorption along the symphysis and mentum osseum.

In AG 3, bone formation dominates the upper face as well as aspects of the midface at the frontal and temporal processes of the zygomatic and on the maxilla at the frontal process and around the nasal aperture. Resorption is concentrated in the lower maxilla, especially the anterior alveolar region, and the anterior half of the zygomatic bone, including the lower orbital margin. The mandible exhibits more extensive resorption in this stage compared to previous stages, with resorption continuing to occur externally on the coronoid process, anterior alveolar region, and symphysis, and internally in the gonial region, ramus, and around the mylohyoid line.

In AG 4, the pattern in the upper and middle face resembles the previous ontogenetic stage (AG 3), except for the zygomatic. The anterior zygomatic is no longer resorptive; rather, resorption occurs in the frontal and temporal process. Resorption also occurs around the glabella. In the mandible, bone formation dominates externally, except at the incisal alveolae, above the mentum osseum, and the posterior alveolar region (around the second/third molar). Internally, resorption persists in the gonial region and to a lesser extent along the ramus and mylohyoid line.

In AG 5, the entire face and mandible are predominantly bone formations. Resorptive regions are generally limited to the lower maxilla, as well as the mandibular incisal alveolae, ramus, and along the mylohyoid line.

### 3.2 Geometric morphometric analysis

**FIGURE 5:**
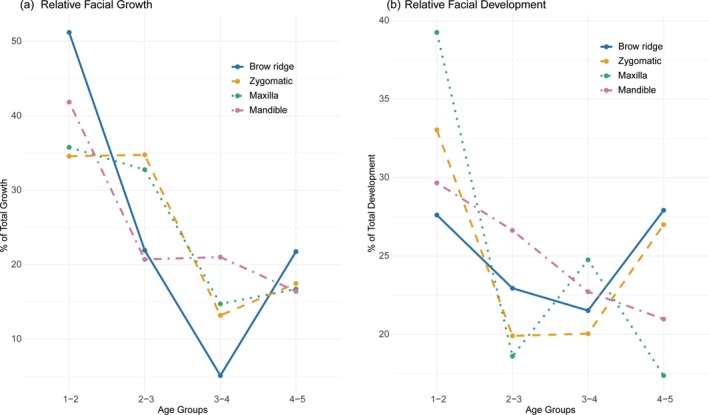
Relative facial growth (a) and development (b) for each facial region measured as growth (centroid size) and developmental (Procrustes coordinates) differences between subsequent age groups.

*Relative amounts of facial growth and development*: The greatest amount of facial growth occurs during early ontogeny, between AG 1 and 2 (Figure 5a). The most notable growth is observed in the brow ridge, with over 50% of total growth occurring between AG 1 and 2. This is followed by the mandible, which shows over 40% of growth occurring between these age groups. For both facial regions, size increases between AG 1 and 2 are significant (*p‐*value: 0.018 and 0.025, respectively; Table 6). The maxilla and zygomatic share a nearly identical pattern of growth with consistent, moderate growth occurring from AG 1 to 3, such that approximately 70% of growth is achieved by the end of AG 3. The mandible follows the maxilla and zygomatic with a small but prolonged growth from AG 2 through 4, and the brow ridge has a distinct growth spurt between AG 4 and 5.

**TABLE 6:** Permutation test results for mean size and shape differences between age groups across facial regions.

Brow ridge	AG 1	AG 2	AG 3	AG 4	AG 5
AG 1	0.000	**0.018**	**0.001**	**0.003**	***p* <0.001**
AG 2	0.201	0.000	0.083	0.161	**0.023**
AG 3	**0.002**	0.165	0.000	0.806	0.141
AG 4	**0.001**	0.065	0.730	0.000	0.335
AG 5	***p* <0.001**	***p* <0.001**	0.072	0.468	0.000

Zygomatic	AG 1	AG 2	AG 3	AG 4	AG 5
AG 1	0.000	0.158	**0.002**	**0.001**	***p* <0.001**
AG 2	**0.008**	0.000	0.066	**0.039**	**0.004**
AG 3	**0.001**	0.171	0.000	0.635	0.223
AG 4	**0.003**	0.319	0.635	0.000	0.564
AG 5	***p* <0.001**	**0.002**	0.309	0.312	0.000

Maxilla	AG 1	AG 2	AG 3	AG 4	AG 5
AG 1	0.000	0.107	**0.002**	**0.001**	***p* <0.001**
AG 2	**0.007**	0.000	0.064	**0.025**	**0.003**
AG 3	***p* <0.001**	0.345	0.000	0.551	0.173
AG 4	***p* <0.001**	**0.025**	0.287	0.000	0.538
AG 5	***p* <0.001**	**0.017**	0.199	0.923	0.000

Mandible	AG 1	AG 2	AG 3	AG 4	AG 5
AG 1	0.000	**0.026**	**0.001**	***p* <0.001**	***p* <0001**
AG 2	0.060	0.000	0.166	**0.024**	***p* <0.001**
AG 3	**0.002**	**0.011**	0.000	0.281	**0.028**
AG 4	**0.001**	**0.013**	0.281	0.000	0.462
AG 5	*p* **<0.001**	*p* **<0.001**	**0.007**	0.430	0.000

*Note*: The upper triangle presents growth differences based on centroid size, while the lower triangle shows developmental differences using Procrustes shape coordinates. Significant values (*p* <0.05) are highlighted in bold.

The greatest amount of facial development also occurs during early ontogeny, between AG 1 and 2 (Figure 5b). Developmental changes are only significant in the maxilla and zygomatic between AG 1 and 2 (*p‐*value: 0.008 and 0.007, respectively; Table 6). The brow ridge and zygomatic exhibit a similar developmental trajectory, with the most pronounced shape changes occurring during early ontogeny (AG 1 and 2) and again in late ontogeny (AG 4 and 5). In contrast, the mandible shows progressively smaller developmental changes between consecutive age groups. The maxilla displays some developmental changes between AG 3 and 4.

**FIGURE 6:**
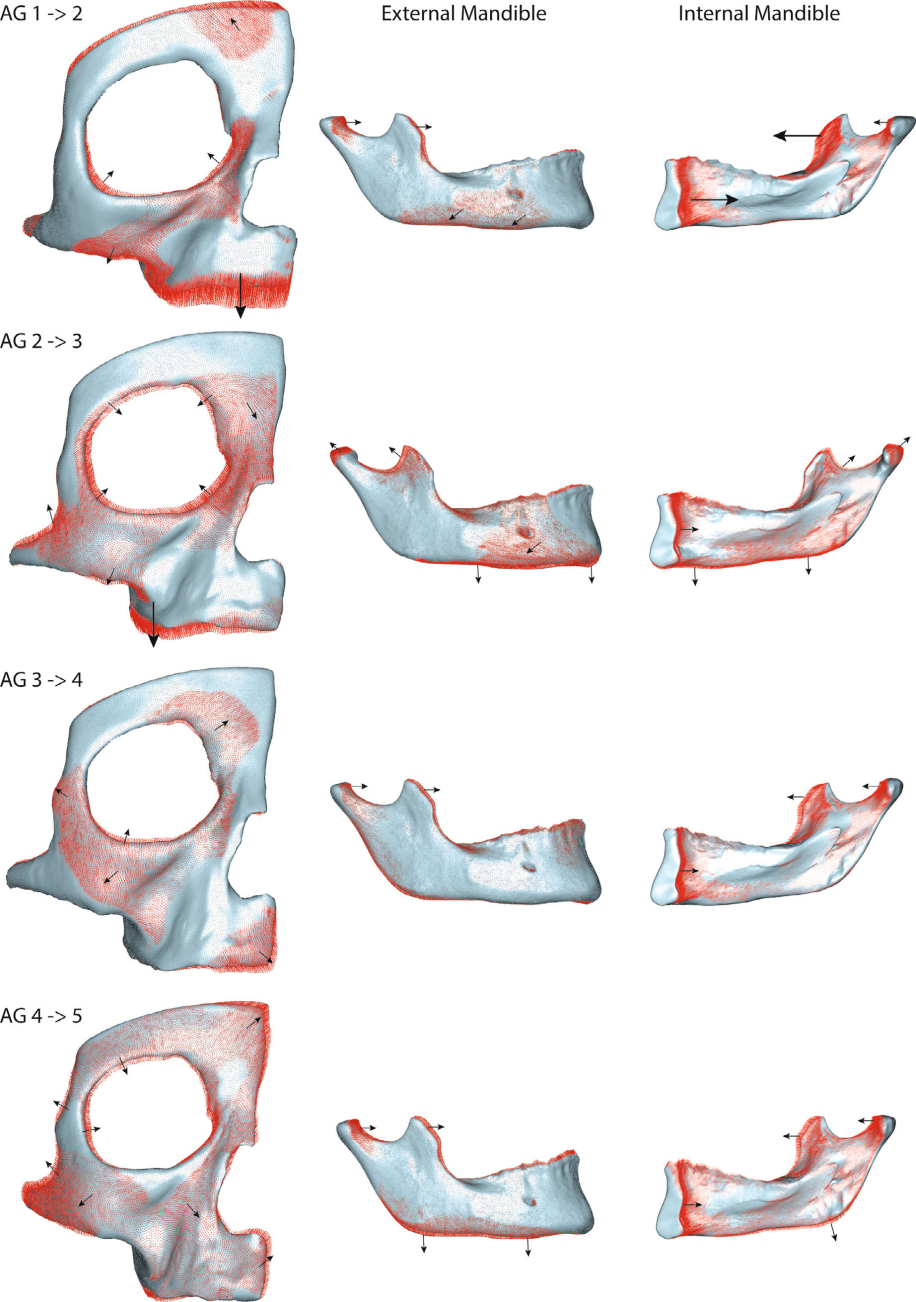
Developmental shape changes during ontogeny. Blue surface is the mean shape for the younger age group and red vectors are the shape differences between it and the subsequent age group (older age group). The longer the vector the greater the shape difference. Black arrows indicate direction of development with larger arrows corresponding to greater development.

*Visualization of developmental shape changes during ontogeny*: From AG 1 to 2, the most prominent shape changes occur in the maxilla, where it increases in size by expanding in a downward direction (Figure 6). Meanwhile, the brow ridge and zygomatic grow upward and backward. Additional notable regions of development occur along the zygomaxillary suture, malar root, and the frontal process of the maxilla. In the mandible, shape changes mainly occur along the anterior ramus and condyle, showing a forward displacement, and internally at the symphysis, showing a backward displacement. This general pattern of development continues throughout ontogeny in the mandible.

From AG 2 to 3, the maxilla continues to grow downward, the zygomatic expands in all directions, the upper orbital margin develops inferiorly, and the interorbital region and maxillary frontal process develop anteriorly. In the mandible, the coronoid process, condyle, and ramus develop superiorly and posteriorly. The internal surface of the corpus shows a backward displacement, and the development of the chin is accentuated.

From AG 3 to 4, the anterior maxilla develops forward and downward, and notable shape changes occur in the anterior zygomatic and frontal process. Shape changes in the mandible are similar to the previous stages but less pronounced.

From AG 4 to 5, the entire upper face expands in a forward and upward direction, and the mandible continues to expand in a forward and downward direction.

**FIGURE 7:**
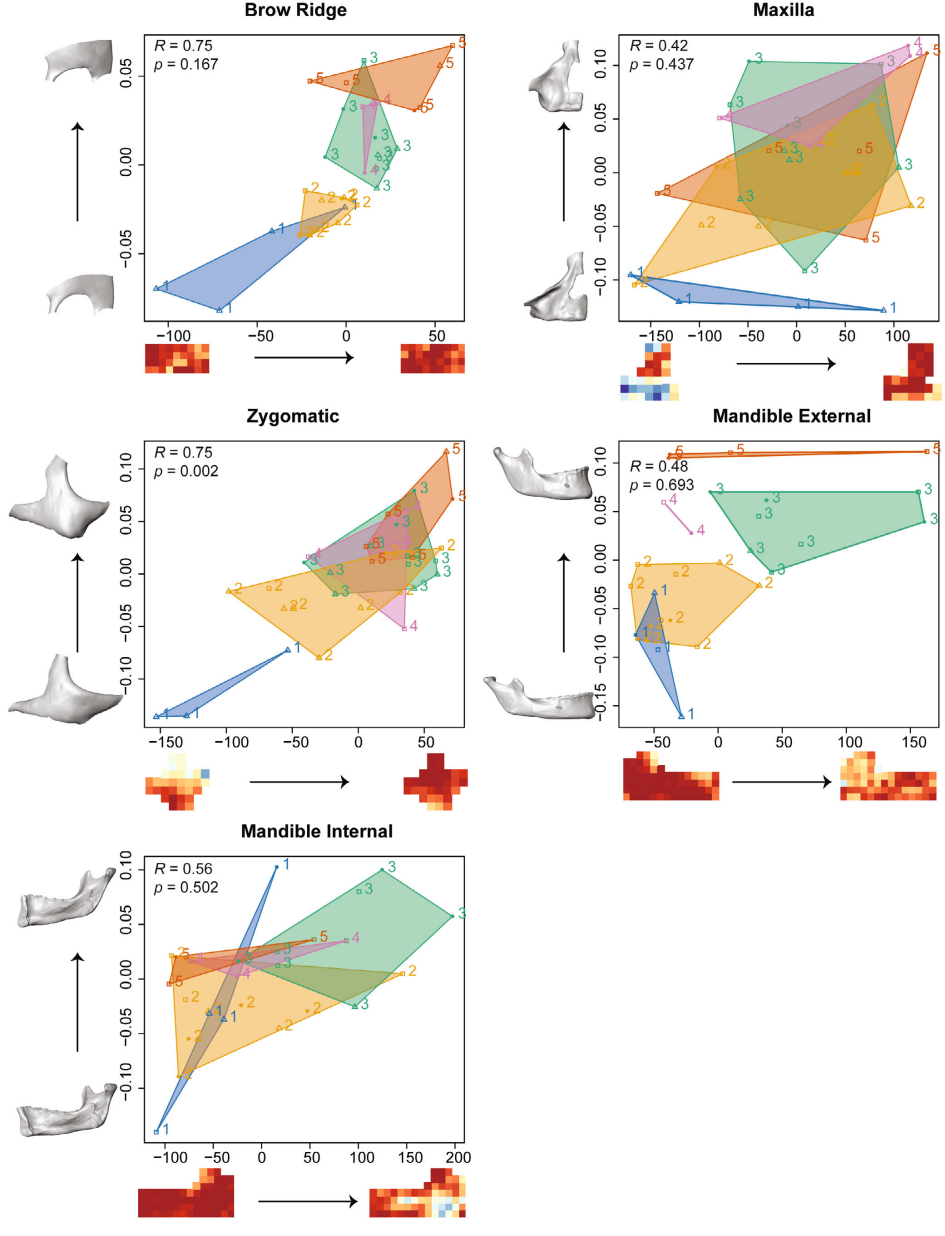
Two‐block partial least squares analysis between bone modeling and Procrustes shape coordinates for each facial region. First singular warp plotted. High percentages of bone resorption (%BR) are shown in cold tones, while warm tones represent low %BR. Convex hulls made for each age group. Extreme shapes and textures (i.e., maps) on each axis warped ± two standard deviations from the mean.

*Relationship between bone modeling and facial development*: Two‐block PLS analyses illustrate the covariation patterns for the first pair of singular warps (SW 1) between the bone modeling and shape analysis for each bone (Figure 7, Table 7). Covariation is only significant for the zygomatic bone (correlation coefficient: 0.75; *p*‐value: 0.002). As the body of the zygomatic lengthens in the supero‐inferior direction and the frontal process widens, the superior portion of the zygomatic (frontal process and orbital margin) shifts from being resorptive in early ontogeny to depository in later ontogeny. All other regions of the face, except the internal mandible, also exhibit clear, but not significant, patterns of covariation during ontogeny. The maxilla and brow ridge become more depository throughout ontogeny. Although quite variable, the pattern of covariation in the maxilla indicates that as maxillary height increases during ontogeny, resorption decreases in the inferior maxilla. As the brow ridge becomes more projecting during ontogeny, areas of bone formation are more extensive. Whereas as the external mandible expands in all directions during ontogeny, resorption extends. Internally, however, the pattern of covariation in the mandible appears to be unrelated to ontogeny. A more vertical ramus and anteriorly inclined symphysis are associated with bone formation. Covariation was significant for several higher pairs of SW, but the percentage of explained total covariance was very low (maxilla: SW 15, 0.13% total covariance; internal mandible: SW 6, 4% total covariance; and brow ridge SW 6, 4.2%, SW 8, 2.3%, and SW 26, < 1% total covariance).

**TABLE 7:** Partial least squares analysis between bone modeling and procrustes shape coordinates for each facial region; singular value, percentage of total covariance, correlation coefficient, and *p*‐values.

Region	Singular value	% Total covariance	Correlation coefficient (*R*)	*p*‐value
Brow ridge	0.94	48.91	0.75	0.167
Maxilla	2.71	54.29	0.42	0.437
Zygomatic	2.31	64.40	0.75	**0.002**
Mandible external	2.37	54.07	0.48	0.693
Mandible internal	2.56	48.86	0.56	0.502

*Note*: Significant values (*p* < 0.05) are highlighted in bold.

## 4 DISCUSSION

This study quantified and visualized facial growth and development at both micro‐ and macroscopic scales across the periosteal surface of the upper, middle, and lower face using surface histology and GM techniques. It is the first study to quantify resorption in the mandible and brow ridge and among the few to examine resorption in infants (others include Kurihara et al., 1980; Schuh et al., 2019, 2020). By integrating these methods and comparing ontogenetic patterns between facial regions, we provide a holistic framework for interpreting the growth and development of facial morphology and for guiding future research.

This study builds upon previous research by incorporating larger sample sizes, as many prior studies analyzed fewer than 25 individuals (e.g., Bromage, 1989; Martinez‐Maza et al., 2013; McCollum, 2008). While our sample improves upon these limitations, we acknowledge that certain geographic groups remain underrepresented in specific age groups. This is particularly relevant for growth and development comparisons between AG 1 and 2, where Western European individuals are predominant. Although efforts were made to ensure balanced representation across age groups and populations, these limitations should be considered when interpreting patterns of facial growth and resorption. Therefore, our findings should be viewed as a foundation for future research.

We show that a greater expression of bone resorption corresponds to significant facial growth and development, particularly in the midface, where high bone turnover likely reflects functional demands related to the oral and nasal cavities. Distinct differences in the expression and pattern of bone resorption and formation occur between the upper, middle, and lower face, reflecting modularity between facial regions (Brachetta‐Aporta et al., 2019b). The upper and lower face primarily exhibit bone formation, whereas the middle face shows greater resorption and variability. Together with previous studies (e.g., Brachetta‐Aporta et al., 2021; Enlow & Hans, 2008; Martinez‐Maza et al., 2013), our findings highlight a common sequence of resorption and deposition associated with facial growth during ontogeny. In general, bone resorption decreases during ontogeny and there is a clear pattern of covariation between bone modeling and shape for most facial regions, except the internal mandible. We also identify a developmental shift in mid‐ontogeny (around AG 3) at both the micro‐ and macroscale marking a transition in upper and midfacial growth, from primarily downward to more forward‐oriented (Martinez‐Maza et al., 2013).

### 4.1 Expression of bone resorption and temporal growth patterns

In line with previous studies, the maxilla is characterized by extensive bone resorption during early ontogeny and more extensive deposition in later ontogeny and adulthood that is associated with greater maxillary height (Brachetta‐Aporta et al., 2021; Enlow & Bang, 1965; Kurihara et al., 1980; Martinez‐Maza et al., 2013; Schuh et al., 2019, 2020). Our results indicate that the maxilla is significantly more resorptive (Table 5) than all other facial regions, reaching maximum resorption in AG 2 (which corresponds to between approximately 8–24 months of age) and continuing with high amounts of resorption until adulthood (AG 5). A greater expression of maxillary resorption in early ontogeny is related to significant amounts of facial growth and development during this age range (AG 1 and 2; Figure 5).

In contrast to the midface, the upper and lower face exhibit more bone deposition overall (Figure 4). The mandible has the least amount of resorption, an unexpected finding given its functional integration with the maxilla (Table 4). Despite these differences, the maxilla and mandible share a similar temporal pattern in their expression of bone resorption. In the mandible, the greatest amount of resorption occurs during AG 3 (between 4.5 and 6.5 years of age), slightly later than the maxilla, and corresponds with substantial mandibular growth (Figure 5). This finding is consistent with Kurihara et al. (1980) who also found mandibular resorption to occur later in development compared to the maxilla. Together, these findings suggest that the delayed onset of resorption in the mandible is a compensatory mechanism reacting to the growth and displacement of the faster growing middle and upper face (Kurihara et al., 1980).

Zygomatic resorption is greatest in the youngest age group (AG 1), corresponding to ages ranging from birth to 8 months. Brow ridge resorption is also highest in AG 1, as well as AG 5 (adulthood). Like the maxilla and mandible, the zygomatic and brow ridge share a similar temporal pattern in the expression of bone resorption, with resorption amounts progressively decreasing in early ontogeny and then increasing again during and following adolescence. When maxillary and mandibular resorption is high, the brow ridge and zygomatic generally express greater bone deposition. This interplay suggests that maxillary resorption facilitates the expansion of the brow ridge and zygomatic, especially in early and late ontogeny. Our GM analyses demonstrate that the zygomatic shares a similar pattern of relative growth with the maxilla and a similar pattern of relative development with the brow ridge; this reflects both the integration of the midface during facial growth and the shared development of sexual dimorphism in the middle and upper face in later ontogeny.

Periosteal bone resorption has only been quantified in a few studies, and only for the maxilla and zygomatic bones. Poor surface preservation in archeological material remains a significant limitation, constraining sample sizes in studies of this kind. To address this issue, we pooled populations in our analyses, which may have obscured some population‐level variation in postnatal development. Schuh et al. (2020) conducted a study on the maxilla to investigate differences in bone modeling patterns between the same populations examined in this study. They observed subtle differences in resorption locations, but the overall pattern and expression of resorption were largely consistent across populations. This suggests that morphological differences between these populations, possibly related to climatic adaptations or subsistence strategy, did not influence bone modeling activity on the periosteal surface. Brachetta‐Aporta et al. (2021) quantified midfacial resorption (maxillary and zygomatic) in two Amerindian populations from Argentina, a horticulturist population from Pampa Grande and a hunter‐gatherer population from Chubut Valley. Overall, they found midfacial resorption to be greater in the hunter‐gatherer population, and, like our study, greater in the maxilla compared to the zygomatic. Both groups exhibited predominant bone resorption in the maxilla slightly later in ontogeny, between the ages of 4.5 and 14.4 years. According to Brachetta‐Aporta and Toro‐Ibacache (2021), who simulated the impact of mechanical loading in these two groups using finite element analysis (FEA), the increased resorption observed in hunter‐gatherers may be attributed to greater mechanical strain associated with their diet. They hypothesized, based on their FEA model, that greater compression strains correspond to resorption activity resulting in fossae and pitting—while tension loading is associated with formation activity, leading to cresting and tuberosity.

Together these studies suggest that the overall temporal pattern of midfacial resorption is similar between diverse populations, being greatest in early ontogeny, but the expression of resorption is variable both within and between populations throughout ontogeny. Greater bone turnover may be due to the central position of the maxilla and zygomatic in the skull and its complex integration with the growth of the surrounding bones and soft tissues not only related to mastication, but also vision, respiration, and sexual dimorphism (Moss & Young, 1960). Additional research that more directly connects function to the studied population is necessary to accurately assess the impact of diet and climate on bone modeling activity.

### 4.2 Micro‐ and macroscopic patterns of facial development and their covariation

Our results largely align with previous research on human facial growth and development and add to a growing body of research documenting periosteal bone modeling patterns in the face (Brachetta‐Aporta et al., 2019b, 2021; Enlow, 1990; Enlow & Hans, 1996; Kurihara et al., 1980; Martinez‐Maza et al., 2013; McCollum, 2008; Schuh et al., 2020). Together these studies demonstrate a general shared pattern of growth and development in the human face. This pattern is conserved during postnatal ontogeny, being similar from birth to adulthood across populations. Overall, the combined bone modeling and shape analyses suggest that the human facial growth pattern reflects the need to maintain a non‐projecting face while supporting the development of sexually dimorphic traits in later ontogeny. However, there are some key developmental shifts around adolescence, primarily in the middle and lower face worth considering, as well as localized changes related to developing dentition, muscle activity, and the development of human‐specific features, like the canine fossa and chin.

As highlighted in previous studies (Brachetta‐Aporta et al., 2019b; Enlow & Hans, 1996; Martinez‐Maza et al., 2013), the brow ridge is predominately depository during ontogeny (Figure 4). The pattern of growth and development of the brow ridge in early ontogeny (AG 1 and 2) differs from the pattern in later ontogeny (AG 3–5). This is reflected in the PLS (Figure 7), which shows a strong (*R* = 0.75), but not significant (*p‐*value: 0.167) pattern of covariation between bone modeling and shape during ontogeny (Table 7). The lack of significance is likely due to small sample sizes. In early ontogeny, the entire brow ridge is more resorptive and beginning around 5 years of age (which corresponds to AG 3 in our sample) it becomes more consistently depository. Morphologically, we show that the brow ridge becomes more projecting with age through a combination of forward growth centrally (i.e., around glabella) and backward growth of the superciliary arches (Figure 6). From AG 4 to 5, there is a shift in the development in the superciliary arches from backward to forward and downward, following the overall forward growth trajectory of the rest of the face during this late ontogenetic stage. This is likely related to the development of sexual dimorphic features during adolescence. Since the brow ridge is predominately bone forming during adolescence and adulthood, sexually dimorphic features, such as the projection of glabella and the lateral brow ridge, appear to develop as a result of differential rates of bone formation; however, a larger sample of individuals of known sex would be needed to further evaluate this.

In agreement with prior studies, the maxilla has the most extensive pattern of resorption and is characterized by greater variability in both bone modeling and shape (Brachetta‐Aporta et al., 2019b, 2021; Enlow & Bang, 1965; Enlow & Hans, 1996; Kurihara et al., 1980; Martinez‐Maza et al., 2013; Schuh et al., 2019; Schuh et al., 2020). Nevertheless, comparisons with these studies suggest that there is a shared bone modeling pattern across populations and during postnatal ontogeny. As already shown by Schuh et al. (2019) and Schuh et al. (2020), the lower maxilla is generally resorptive around the alveolar process and canine fossa, and the upper maxilla is generally depository around the nasal aperture and the frontal process. Bone resorption on the canine fossa begins around AG 2 and remains like that until AG 4; however, morphologically, the canine fossa is present already in the earliest age group. We identify, both micro‐ and macroscopically, a shift in the direction of maxillary growth beginning around adolescence (from AG 3 to 4) from primarily downward to forward. Microscopically, the lower maxilla changes from being largely resorptive during ontogeny to depository in adulthood. Macroscopically, forward growth is initiated via the anterior maxilla in the adolescence (AG 4) stage, followed by a forward growth of the entire bone from adolescence to adulthood (from AG 4 to 5).

The zygomatic also expresses a clear developmental shift around adolescence (from AG 3 to 4) at both the micro‐ and macroscopic levels. This is the only facial region in our study to show a strong and significant (*R* = 0.75, *p‐*value: 0.002) pattern of covariation between bone modeling and shape during ontogeny. This is the first study to show bone resorption on the frontal process of the zygomatic. In previous studies, bone resorption in subadults was limited to the orbital margin (Brachetta‐Aporta et al., 2019b, 2021; Enlow & Hans, 1996; Martinez‐Maza et al., 2013). This is also the first study to document the zygomatic bone modeling pattern in such young individuals. The only other studies with potentially comparably young ages are from Brachetta‐Aporta et al., (2019b, 2021); however, no specific ages are given, and their youngest age group consists of only five individuals that span the age range of 0–4.5 years. Resorption on the frontal process corresponds to a backward growth of the entire upper face, including the frontal process, and accommodates the significant amount of growth that occurs in the upper face from AG 1 to 2. In other respects, the bone modeling pattern for the zygomatic reported in the study is similar to previous studies by showing resorption along the orbital rim throughout ontogeny and along the inferior zygomatic in adulthood (Brachetta‐Aporta et al., 2019b, 2021; Enlow & Hans, 1996; Martinez‐Maza et al., 2013). The frontal and temporal process, often related to facial robusticity and sex estimation (Lahr & Wright, 1996; Oettlé et al., 2017), are generally depository, and their shape becomes more pronounced in late ontogeny. Resorption on the inferior zygomatic body in adults corresponds to the insertion site for the masseter muscles and might be related to that or to compression strains from mastication (Brachetta‐Aporta et al., 2021).

The bone modeling pattern of the mandible is related to facial retraction, developing dentition, and muscle attachment sites. In the youngest ontogenetic stages (AG 1 and 2), bone resorption on the labial corpus is localized around the anterior alveolar margin and symphysis. This pattern is generally maintained until adulthood, at which point the anterior corpus becomes more depository, except around the labial incisal region. This pattern is in general agreement with previous studies, but with some deviations. Kurihara et al. (1980) describe a bone modeling reversal in early ontogeny that is not apparent in our study. They found the labial side of the symphysis to be depository until the emergence of the second deciduous molar (around 2 years of age), followed by resorption, beginning at the incisal alveolar margin and spreading downward, variably covering the entire symphyseal region by the time the first permanent molar erupts. This bone modeling pattern describes the development of the chin, where the inferior portion of the symphysis grows forward from deposition in early ontogeny and the bone above it (around the roots of the incisors) is resorbed, leaving behind a projecting shelf of bone. This pattern is also evident in our study, especially in later ontogeny (e.g., AG 4), but unlike Kurihara et al. (1980), we identify resorption in the anterior corpus already in our youngest age group (between birth and 8 months). We also show that symphyseal growth is accomplished lingually through bone deposition, resulting in a backward growth of the internal symphysis. Together, our bone modeling and shape analyses demonstrate the progressive development of the human chin throughout ontogeny, aligning with previous studies showing that the mental eminence begins to form before the emergence of the first permanent molar (Ackermann & Krovitz, 2002; Bulygina et al., 2006; Coquerelle et al., 2010).

The bone modeling pattern of the anterior corpus described in this study differs slightly from Martinez‐Maza et al. (2013), who found it to be more depository in subadult individuals from 8 to 12 years of age. This corresponds to AG 4 in our study, an age group that displays pronounced resorption on the anterior corpus. Resorption on the labial surface of the anterior corpus is related to both deciduous and permanent anterior tooth reorientation and to maintain structural alignment with the maxilla. Enlow's counterpart principle (1990) asserts that the anterior corpus follows the displacement of the ethmo‐maxillary complex to allow for occlusion of the teeth. This principle is most evident during AG 3, when resorption is most extensive on the mandible, following extensive maxillary resorption in the previous age group (AG 2).

Our findings on the lingual corpus align with Martinez‐Maza et al. (2013), who observed resorption fields around the mylohyoid line and sublingual fossa. This resorption is likely associated with the development of the adult postcanine dentition, which emerges later in ontogeny around AG 3 and continues into adulthood. Our shape analysis further supports this process, showing a deepening of the fossa. Notably, Enlow and Hans (2008) did not identify these resorption fields.

The bone modeling pattern observed on the mandibular ramus aligns with previous research with resorption along the anterior portion of the ramus and deposition along the posterior border, gradually shifting the entire ramus posteriorly (Enlow & Hans, 2008; Hans et al., 1995; Martinez‐Maza et al., 2013). This pattern explains the vertical repositioning of the ramus with age (Terhune et al., 2014). We also identified resorption fields at the coronoid process buccally and the gonial region lingually in early ontogeny (from AG 1 to 3). These resorption fields correspond to muscle attachment sites related to mastication, particularly for the temporalis at the coronoid process and the medial pterygoid at the gonial angle. As noted earlier on the zygomatic, these resorption fields may be related to either muscle attachments or compression strains. Interestingly, resorption occurs in these regions (coronoid process and gonial region) before the introduction of solid foods. While these muscles are not yet engaged in chewing, they remain active in other functions, contributing to sucking and early jaw movements essential for breastfeeding.

## 5 CONCLUSION

This study is the first to quantify and visualize facial ontogeny from birth to adulthood across the upper, middle, and lower face using surface histology and geometric morphometrics. By integrating these methods and comparing ontogenetic patterns across facial regions, we provide a comprehensive framework for understanding postnatal facial development. Our findings reveal a strong covariation between bone modeling and shape in most facial regions and demonstrate that key aspects of adult bone modeling patterns and morphology are already established at birth. Resorption is present in early ontogeny, is greatest in the maxilla, and decreases over time. A developmental shift around adolescence marks a transition from primarily downward to more forward‐oriented growth. The postnatal bone modeling pattern in humans reflects the need to maintain a non‐projecting face, while resorption also facilitates localized changes associated with developing dentition, muscle attachment sites, and human‐specific traits such as the canine fossa and mental eminence.

Poor surface preservation in archaological specimens remains a significant limitation, constraining sample sizes in studies of this kind. To address this issue, we pooled populations in our analysis, which may have obscured meaningful population‐level variation in postnatal development. Additionally, some geographic groups are underrepresented in several age groups, potentially confounding our interpretations. Expanding sample sizes and incorporating individuals with known sex in future studies will provide deeper insights into the role of these factors in facial growth and may improve age and sex estimations in both past and present human populations.

This study lays a foundation for future research on bone modeling in fossil hominins, enabling more comprehensive comparisons across species. Bone modeling studies on fossil hominins remain limited and primarily focused on the maxilla. Research on Plio‐Pleistocene African hominins has shown that their maxillae are largely characterized by bone formation, suggesting that bone deposition is likely the ancestral condition (Bromage, 1989; Lacruz, Bromage, O'Higgins, Toro‐Ibacache, et al., 2015; McCollum, 2008). The earliest potential evidence so far of modern human‐like maxillary resorption has been identified on ATD 6–69, a juvenile *Homo antecessor* maxilla dated to approximately 850,000 (Lacruz et al., 2013), though poor surface preservation of this fossil makes this interpretation uncertain. In Neanderthals, extensive bone formation on the maxillary surface has been linked to midfacial prognathism (Lacruz, Bromage, O'Higgins, Arsuaga, et al., 2015; Schuh et al., 2025). Studies on several pre‐Neanderthal mandibles from Sima de los Huesos and Neanderthals from El Sidrón indicate differences in bone modeling patterns compared to recent humans (Martinez‐Maza et al., 2011; Rosas & Martinez‐Maza, 2010). Given their distinct zygomatic morphology, Neanderthals likely exhibited unique bone modeling patterns in this region. Additionally, much remains unknown about the facial growth patterns of the earliest members of our species, whose facial skeletons were larger and more robust than those of recent humans yet less prognathic than Neanderthals.

By expanding our understanding of postnatal facial ontogeny in recent humans, this study provides a critical framework for future comparative research on fossil hominins. Further investigations incorporating larger, more diverse samples will be essential for unraveling the evolutionary and developmental processes shaping human facial morphology.

## AUTHOR CONTRIBUTIONS

**Sarah E. Freidline:** Conceptualization; investigation; writing – original draft; methodology; visualization; writing – review and editing; formal analysis; supervision; data curation; project administration. **Madison Hubbart:** Writing – review and editing; methodology. **Catherine Shipman:** Methodology; writing – review and editing. **Najielie Burgos:** Methodology; writing – review and editing. **Chiara Villa:** Resources; writing – review and editing. **Alexandra Schuh:** Writing – review and editing; methodology; formal analysis; conceptualization; investigation; visualization.

## CONFLICT OF INTEREST STATEMENT

The author(s) declare no competing interests.

## Supporting information


**Supplementary Figure 1.** Landmarks (red), curve semilandmarks (blue), and surface semilandmarks (yellow) used in the geometric morphometric analyses. Landmarks subsets include: (a) brow ridge; (b) zygomatic; (c) maxilla; (d) external mandible; and (e) internal mandible. Landmark and curve definitions provided in Table 3.

**Supplementary Table 1:** Number of individuals attributed to each age group (AG) for each population in the bone modeling analysis.
**Supplementary Table 2**: Number of individuals attributed to each age group (AG) for each population in the geometric morphometric analyses. The (semi)landmark dataset for the mid and upper face includes the brow ridge, maxilla, and zygomatic, and the (semi)landmark dataset for the mandible includes both the internal and external sides.
