# Supplementary figures and images for: Shaping the human face: Periosteal bone modeling across ontogeny
Source: Anat Rec (Hoboken). 2025 May 19;309(2):271–92. doi: 10.1002/ar.25689 (PMC12803535; doi:10.1002/ar.25689)

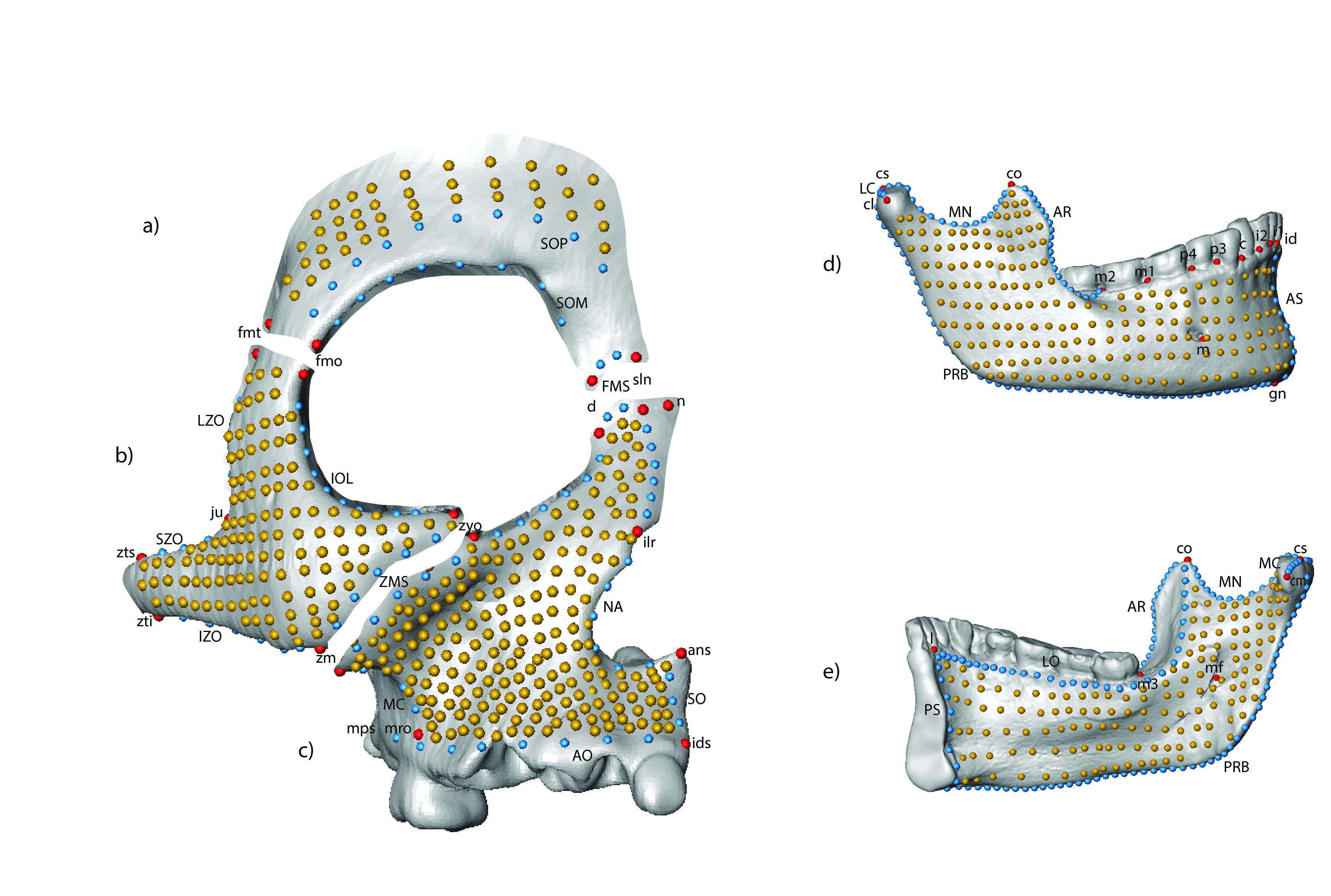

Supplement: Supplementary file 1 — Supplementary Figure 1. Landmarks (red), curve semilandmarks (blue), and surface semilandmarks (yellow) used in the geometric morphometric analyses. Landmarks subsets include: (a) brow ridge; (b) zygomatic; (c) maxilla; (d) external mandible; and (e) internal mandible. Landmark and curve definitions provided in Table 3. [file AR-309-271-s001.tif]
