# Supplementary material for: Shaping the human face: Periosteal bone modeling across ontogeny
Source: Anat Rec (Hoboken). 2025 May 19;309(2):271–92. doi: 10.1002/ar.25689 (PMC12803535; doi:10.1002/ar.25689)
Supplement: Supplementary file 2 — Supplementary Table 1: Number of individuals attributed to each age group (AG) for each population in the bone modeling analysis. Supplementary Table 2: Number of individuals attributed to each age group (AG) for each population in the geometric morphometric analyses. The (semi)landmark dataset for the mid and upper face includes the brow ridge, maxilla, and zygomatic, and the (semi)landmark dataset for the mandible includes both the internal and external sides. [file AR-309-271-s002.docx]

**Supplementary Table 1**: Number of individuals attributed to each age group (AG) for each population in the bone modeling analysis.

| **Population** | **AG** | **Brow ridge** | **Maxilla** | **Zygomatic** | **Mandible External** | **Mandible Internal** |
| --- | --- | --- | --- | --- | --- | --- |
| Khoe/San | 1 | 1 | 0 | 1 | 1 | 1 |
|  | 2 | 5 | 7 | 5 | 4 | 4 |
|  | 3 | 3 | 4 | 4 | 4 | 3 |
|  | 4 | 2 | 2 | 2 | 1 | 2 |
|  | 5 | 4 | 4 | 4 | 2 | 2 |
| Total |  | 15 | 17 | 16 | 12 | 12 |
| Western European | 1 | 4 | 4 | 3 | 4 | 4 |
|  | 2 | 5 | 8 | 5 | 5 | 5 |
|  | 3 | 4 | 4 | 4 | 4 | 4 |
|  | 4 | 0 | 0 | 0 | 0 | 0 |
|  | 5 | 3 | 2 | 3 | 2 | 2 |
| Total |  | 16 | 18 | 15 | 15 | 15 |
| Greenlandic Inuit | 1 | 0 | 0 | 0 | 2 | 2 |
|  | 2 | 5 | 5 | 5 | 4 | 4 |
|  | 3 | 6 | 6 | 5 | 3 | 3 |
|  | 4 | 2 | 3 | 3 | 1 | 1 |
|  | 5 | 2 | 2 | 2 | 2 | 2 |
| Total |  | 15 | 16 | 15 | 12 | 12 |

**Supplementary Table 2**: Number of individuals attributed to each age group (AG) for each population in the geometric morphometric analyses. The (semi)landmark dataset for the mid and upper face includes the brow ridge, maxilla, and zygomatic, and the (semi)landmark dataset for the mandible includes both the internal and external sides.

| **Population** | **AG** | **Mid/Upper Face** | **Lower Face** |
| --- | --- | --- | --- |
| Khoe/San | 1 | 0 | 1 |
|  | 2 | 3 | 4 |
|  | 3 | 3 | 4 |
|  | 4 | 2 | 2 |
|  | 5 | 4 | 4 |
| Total |  | 12 | 14 |
| Western European | 1 | 4 | 2 |
|  | 2 | 8 | 5 |
|  | 3 | 4 | 1 |
|  | 4 | 0 | 0 |
|  | 5 | 0 | 0 |
| Total |  | 12 | 6 |
| Greenlandic Inuit | 1 | 0 | 1 |
|  | 2 | 2 | 4 |
|  | 3 | 2 | 5 |
|  | 4 | 2 | 3 |
|  | 5 | 1 | 2 |
| Total |  | 7 | 15 |
